# Prone position: how understanding and clinical application of a technique progress with time

**DOI:** 10.1007/s44254-022-00002-2

**Published:** 2023-03-09

**Authors:** Luciano Gattinoni, Serena Brusatori, Rosanna D’Albo, Roberta Maj, Mara Velati, Carmelo Zinnato, Simone Gattarello, Fabio Lombardo, Isabella Fratti, Federica Romitti, Leif Saager, Luigi Camporota, Mattia Busana

**Affiliations:** 1 Department of Anesthesiology, University Medical Center Göttingen, Robert Koch Straße 40, 37075 Göttingen, Germany; 2 IRCCS San Raffaele Scientific Institute, Milan, Italy; 3 Department of Adult Critical Care, Guy’s and St Thomas’ NHS Foundation Trust, Health Centre for Human and Applied Physiological Sciences, London, UK

**Keywords:** Prone position, Gas exchange, Lung mechanics, ARDS, Covid-19

## Abstract

**Historical background:**

The prone position was first proposed on theoretical background in 1974 (more advantageous distribution of mechanical ventilation). The first clinical report on 5 ARDS patients in 1976 showed remarkable improvement of oxygenation after pronation.

**Pathophysiology:**

The findings in CT scans enhanced the use of prone position in ARDS patients. The main mechanism of the improved gas exchange seen in the prone position is nowadays attributed to a dorsal ventilatory recruitment, with a substantially unchanged distribution of perfusion. Regardless of the gas exchange, the primary effect of the prone position is a more homogenous distribution of ventilation, stress and strain, with similar size of pulmonary units in dorsal and ventral regions. In contrast, in the supine position the ventral regions are more expanded compared with the dorsal regions, which leads to greater ventral stress and strain, induced by mechanical ventilation*.*

**Outcome in ARDS:**

The number of clinical studies paralleled the evolution of the pathophysiological understanding. The first two clinical trials in 2001 and 2004 were based on the hypothesis that better oxygenation would lead to a better survival and the studies were more focused on gas exchange than on lung mechanics. The equations better oxygenation = better survival was disproved by these and other larger trials (ARMA trial). However, the first studies provided signals that some survival advantages were possible in a more severe ARDS, where both oxygenation and lung mechanics were impaired. The PROSEVA trial finally showed the benefits of prone position on mortality supporting the thesis that the clinical advantages of prone position, instead of improved gas exchange, were mainly due to a less harmful mechanical ventilation and better distribution of stress and strain. In less severe ARDS, in spite of a better gas exchange, reduced mechanical stress and strain, and improved oxygenation, prone position was ineffective on outcome.

**Prone position and COVID-19:**

The mechanisms of oxygenation impairment in early COVID-19 are different than in typical ARDS and relate more on perfusion alteration than on alveolar consolidation/collapse, which are minimal in the early phase. Bronchial shunt may also contribute to the early COVID-19 hypoxemia. Therefore, in this phase, the oxygenation improvement in prone position is due to a better matching of local ventilation and perfusion, primarily caused by the perfusion component. Unfortunately, the conditions for improved outcomes, i.e. a better distribution of stress and strain, are almost absent in this phase of COVID-19 disease, as the lung parenchyma is nearly fully inflated. Due to some contradictory results, further studies are needed to better investigate the effect of prone position on outcome in COVID-19 patients.

**Graphical Abstract:**

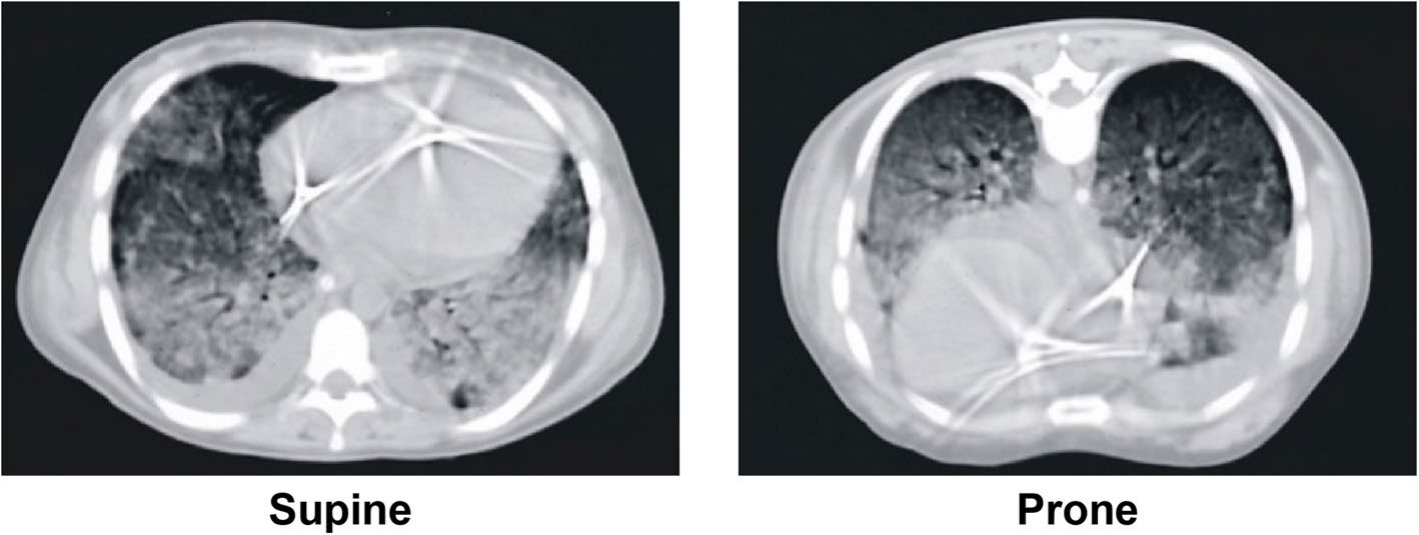

## Historical background

In 1974, Charles Bryan was the first to propose, on a theoretical basis, to keep in “serious consideration” the use of prone position to improve ventilation of the dorsal lung regions [1]. His proposal was based on his previous study on healthy supine paralyzed patients during anesthesia where it was observed that, due to the interaction with the abdominal content, the dorsal regions where hypoventilated compared to the ventral ones. Two years later, Piehl et al. [2] applied prone position to five patients with Acute Respiratory Distress Syndrome (ARDS) and reported an impressive improvement of oxygenation compared to when they were supine. Similar observations were made by Douglas et al. [3] who reported oxygenation improvement in prone position in six patients with ARDS. Of note, however, these considerations and observations had little impact on the critical care community. The reasons are not clear, but likely depend on the exiguous number of patients studied and on the logistic difficulties to place and keep the patients in prone position. In addition, there were all the practical issues linked to the prone decubitus, including the impossibility to perform emergency interventions, the development of pressure ulcers, the risk of devices displacement, the fear of providing enteral nutrition, etc. In short, it is likely that the game was not considered worth the candle.

**Fig. 1:**
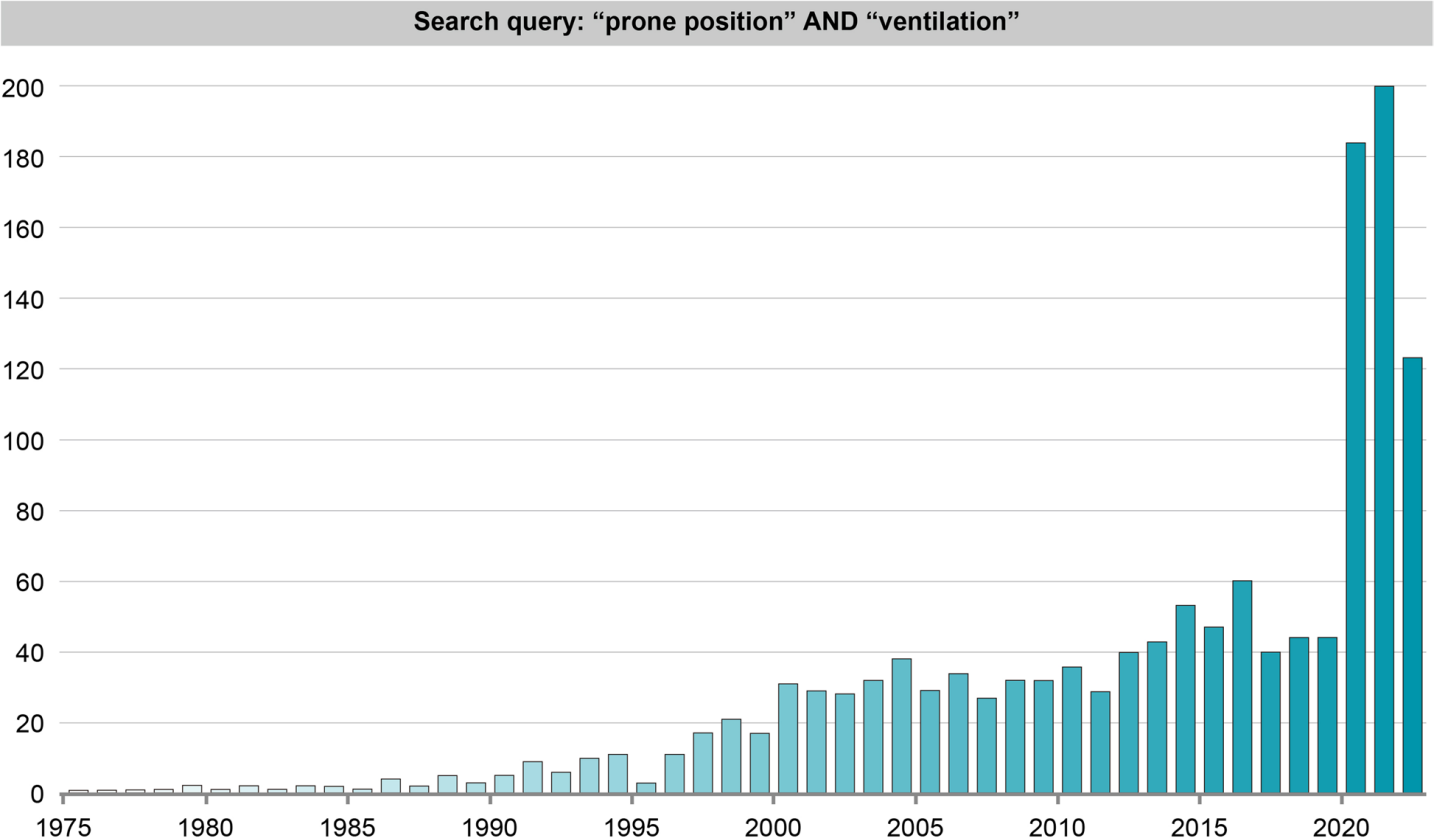
Number of studies per year about “prone position” and “ventilation”. The research was conducted in PubMed, suing as search query: “prone position” AND “ventilation”

However, in May 1986, two studies independently published reported the anatomy of ARDS as described by CT-scan [4, 5]. Both studies showed that the densities were not homogeneously distributed throughout the lung parenchyma, but primarily concentrated in the dependent lung regions. These observations led us to prone patients assuming that the ventral regions of the lung could be better perfused in prone position, thus justifying the oxygenation improvement seen in most of patients. However, when we performed CT-scans in prone position, we found that the lung densities redistributed with the change in position from dorsal to ventral zones [6, 7], thus disproving the hypothesis that the primary mechanism of oxygenation improvement was the change in regional perfusion. One year later, Albert et al. [8] confirmed the oxygenation improvement in experimental animals, but the mechanism was still elusive, as functional residual capacity (FRC) and lung mechanics, as well as the diaphragm position were unmodified in prone position despite improvement in oxygenation. Since then, the number of studies on ventilation in prone position progressively increased year on year (Fig. 1), until the latest “explosion” of the use of prone position during the COVID-19 pandemic. We believe that the prone position may be considered a model on how the clinical medicine may progress. Indeed, over the years, it has been a continuous exchange between physiological, observational studies and randomized controlled trials (RCT). The initial hypothesis on the diversion of lung perfusion as a cause of oxygenation improvement and the improved oxygenation as a cause of better survival has been rejected in favor of alternative mechanisms of improvement in gas exchange, indications for its use and the effects on survival.

In this review we will discuss those aspects we believe to be more relevant, discussing at the end the prone position in patients with COVID-19, highlighting how this condition is different from the classical ARDS.

## Physiology of prone position

### Gas exchange

#### Oxygenation

**Fig. 2:**
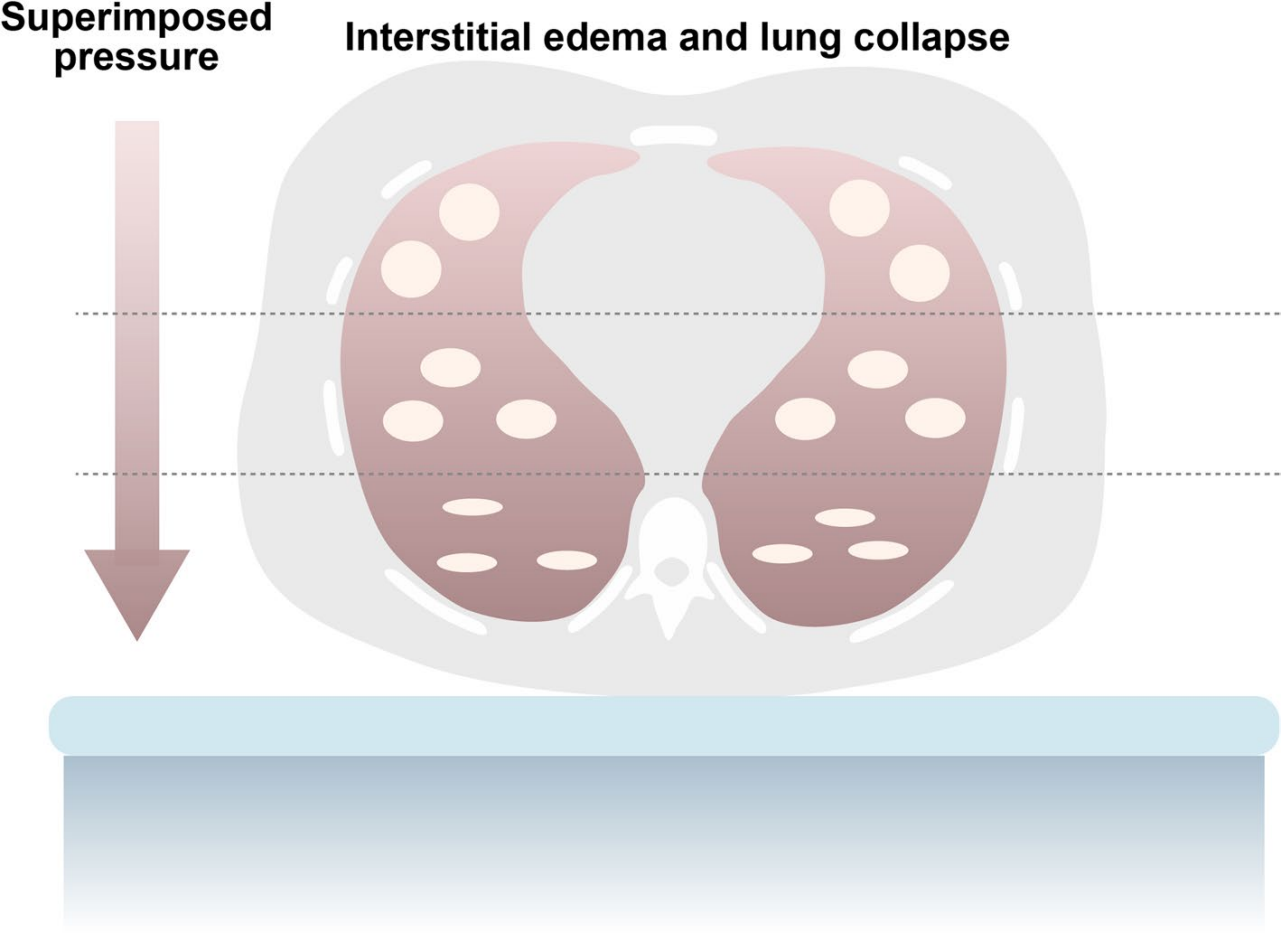
Hydrostatic pressure – The sponge model. The lung of a patient with ARDS, characterized by interstitial edema, can be considered as a wet sponge. According to a gravity-dependent gradient, the edematous lung tissue exerts a hydrostatic pressure over the tissue below, leading to lung collapse in the dependent regions

To better understand the physiological modifications occurring during prone position in patients with ARDS, it is worth to starting from the pathophysiological features of this syndrome. ARDS is a life-threatening condition, in which the primary pathological root is an acute inflammatory lung injury, where the increased pulmonary vascular permeability leads to the development of lung edema and atelectasis [9]. A typical radiological feature of the ARDS lung in supine position is a progressive increase in lung density from the sternum to the vertebral column, which corresponds to a progressive decrease in the percentage of aerated lung tissue [4, 10]. This has been well explained through the idea of a lung as a sponge – “the sponge model” (Fig. 2) [11]. Indeed, the ARDS lung can be compared to a wet sponge, where edema is quite evenly distributed [12]. Because of the hydrostatic pressure progressively exerted by the heavy, edematous lung parenchyma on the tissue underneath, gas is squeezed out from the dorsal regions (*dependent* in supine position), while the ventral *nondependent* regions remain well aerated – the so-called “baby lung” [13, 14]*.* The heart weight contributes to the compression of the dorsal lung parenchyma in supine position [15].

**Fig. 3:**
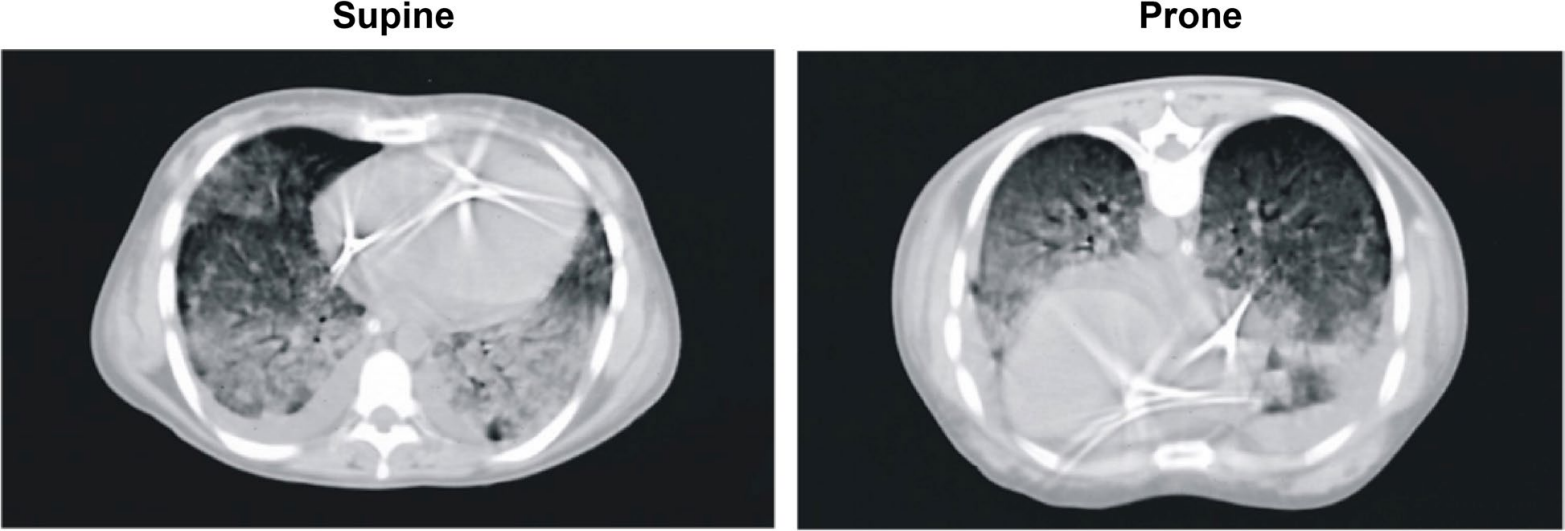
CT-scans in supine *vs.* prone position at end-expiration. Representative CT-scans of a patient with ARDS, showing that lung densities redistribute from the dorsal to ventral regions when moving from supine to prone position

One proposed mechanism to explain the observed oxygenation improvement in prone position was an increase in end-expiratory lung volume [3], but this finding was not confirmed in subsequent experimental [8] and clinical studies [16]. Another possible cause of oxygenation improvement was proposed as an increased gravity-dependent perfusion of the baby lung in prone position, as the baby lung was hypothesized as a fixed anatomical entity located in the dorsal area of the thoracic cage [6]. This hypothesis, however, also proved to be flawed, as CT-scans clearly showed that the baby lung is a functional rather than an anatomical entity [17]. Indeed, after pronation the lung densities redistributed from dorsal to ventral (Fig. 3) [7]. The dorsal regions, no more subjected to the pressure of the superimposed lung, tended to reopen, while the ventral regions tended to collapse under the superimposed pressure. If – with the change in position – the reopening of the dorsal lung tissue is greater than the ventral collapse, a net recruitment is achieved (this occurs in the majority of the patients) and this net recruitment is associated with increased oxygenation if the perfusion of the previously collapsed areas remains unchanged. Actually, this has been confirmed in a series of studies that demonstrated that the pulmonary regional perfusion is substantially gravity independent and that the dorsal regions remain equally perfused in prone and supine, leading to oxygenation improvement if the net recruitment occurs [18–22].

#### CO_2_ clearance

In prone position, the arterial partial pressure of CO_2_ (P_a_CO_2_) could either, decrease, increase or remain unchanged compared to supine, depending on the relative variations in lung perfusion and alveolar ventilation, which must be clearly distinguished from the changes in lung inflation. The P_a_CO_2_ decreases when the net recruitment is the prevailing phenomenon during prone [23]. Indeed, the ventilation is redirected from the ventral regions now collapsed, to the dorsal regions now recruited. If the balance between these two opposite phenomena favors an overall decrease of ventilation/perfusion ratio, the CO_2_ clearance increases and therefore the P_a_CO_2_ decreases. This has been associated to greater survival [24, 25]. In contrast, if poor alveolar recruitment occurs (*i.e*., the dorsal recruited pulmonary units are fewer than the ventral collapsed ones), the upright diversion of the ventilation may lead to an increase of the dead space fraction. This may lead to an increase in P_a_CO_2_. When these two phenomena are balanced, the P_a_CO_2_ may remain unmodified between prone and supine position. It is important to understand the possible factors leading to a dissociation between oxygenation and CO_2_ clearance. Indeed, an inflated and perfused pulmonary unit is required to oxygenate the arterial blood. In contrast, if the same inflated unit is hypoventilated, *i.e.* the exchange of gas into the unit is decreased, the CO_2_ is not cleared and we observe an increase in both arterial partial pressure of O_2_ (P_a_O_2_) and P_a_CO_2_.

### Lung mechanics

**Fig. 4:**
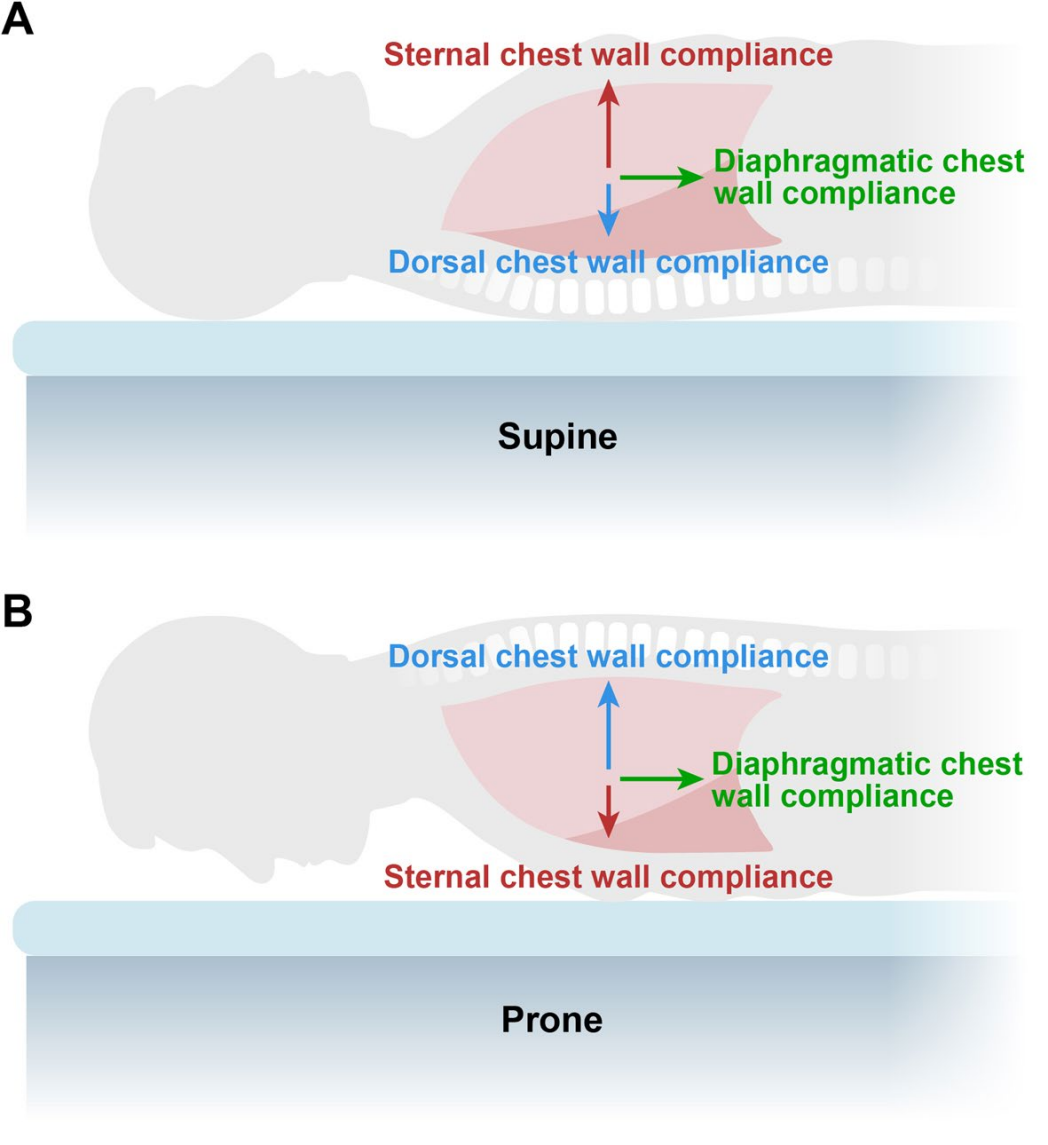
Chest wall compliance is the sum of three components: anterior (sternal), posterior (dorsal) and diaphragmatic. The anterior chest wall is more compliant than the posterior one, due to anatomical reasons. Therefore, in supine position the gas is mainly distributed primarily towards the anterior and diaphragmatic regions and less to the posterior ones. In prone position, the anterior chest wall compliance decreases, the dorsal one increases, though not reaching the values of the sternal compliance in supine. The diaphragmatic compliance is substantially unmodified. Thus, the final result is that in prone position the total respiratory system compliance decreases if the lung compliance remains unmodified

The primary alteration of lung mechanics when shifting from prone to supine is a decrease of chest wall compliance. Because of the anatomical conformation of the rib cage, its ventral part (sternum) is much more deformable than its posterior one (spine). When lying supine, the stiff dorsal part of the rib cage is in contact with the bed, while the deformable ventral part is able to expand (Fig. 4). It follows that most of alveolar ventilation will inflate the ventral rather than the dorsal lung regions. On the other hand, in prone position, chest wall compliance is decreased [16, 26, 27], as only the stiff dorsal part is free to move. Therefore, during prone position, the normal reaction is a decrease in total compliance of the respiratory system, due to the relative increase in the chest wall component. Consequently, when the patient is put in prone position during volume-controlled ventilation, the plateau pressure and the measured compliance will decrease. If the plateau pressure decreases, it indicates that the increased lung volume due to the recruitment – and therefore lung compliance – increases proportionally more than the decrease in the chest wall compliance. If the plateau does not change, it means that the natural decrease of chest wall compliance is compensated by the increase of lung compliance [16]. Another interesting finding, noticed in supine position, is that a manual compression of the chest wall may result in a paradoxical increase of tidal volume during volume-controlled ventilation, or a decrease in plateau pressure during pressure-controlled ventilation [28]. This phenomenon, when present, unmasks the presence of lung hyperinflation, a condition in which the decrease of chest wall compliance by manual compression leads to a reduction in the hyperinflated lung volume, placing the lung in a more favorable position of pressure–volume curve. Hyperinflation, revealed by this maneuver, is often associated with hypercapnia and hypoventilation, as discussed above. In addition, the manual compression of chest wall in supine position partially mimics the chest wall compliance reduction observed in prone, with possible redistribution of ventilation to the less aerated dependent regions. A synchronization between tidal insufflation and chest wall compression has been patented decades ago [29].

**Fig. 5:**
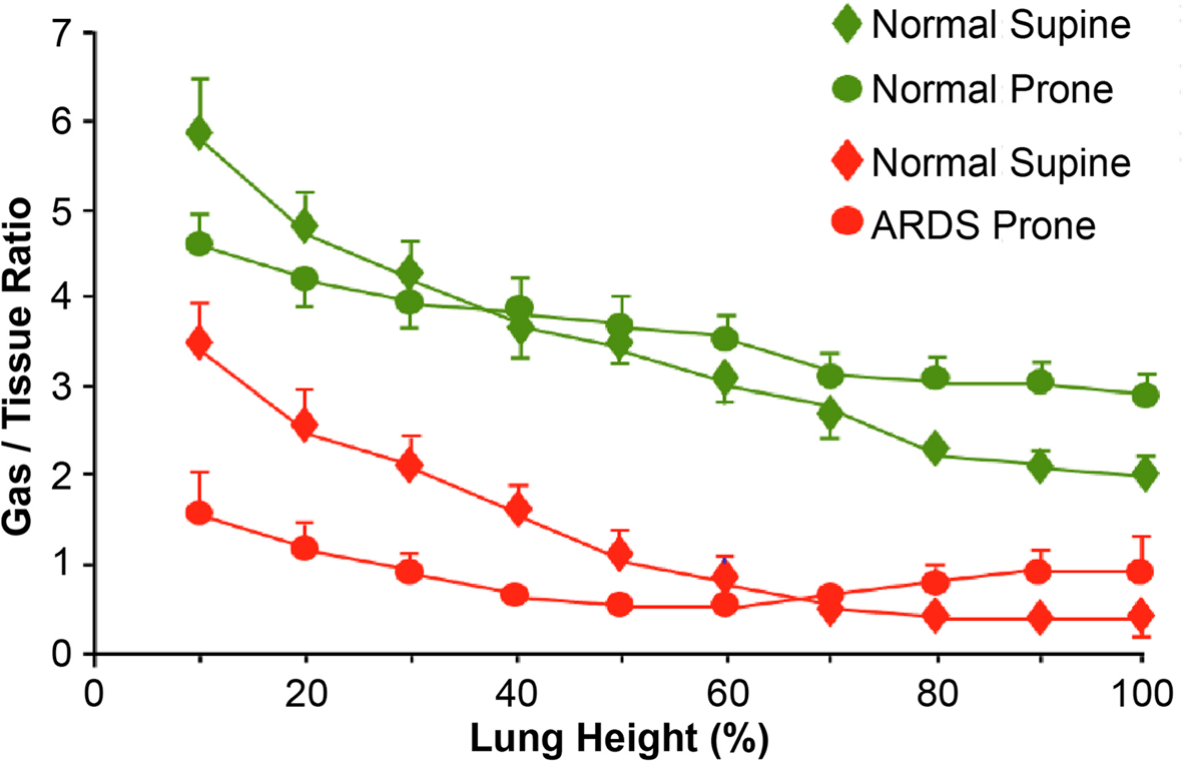
Homogenization of gas/tissue distribution. Representation of gas/tissue ratio, *i.e.* an index of regional lung inflation derived from CT-scans analysis, as a function of lung height in the supine (diamonds) and prone (circles) positions. A height of 0% refers to the nondependent surface of the thorax (ventral in the supine position and dorsal in the prone position). Conversely, a height of 100% refers to the dependent surface of the thorax. The green symbols refer to normal lungs (*n* = 14), while the red symbols to lungs of patients with ARDS (*n* = 20). Adapted from reference [32]

**Fig. 6:**
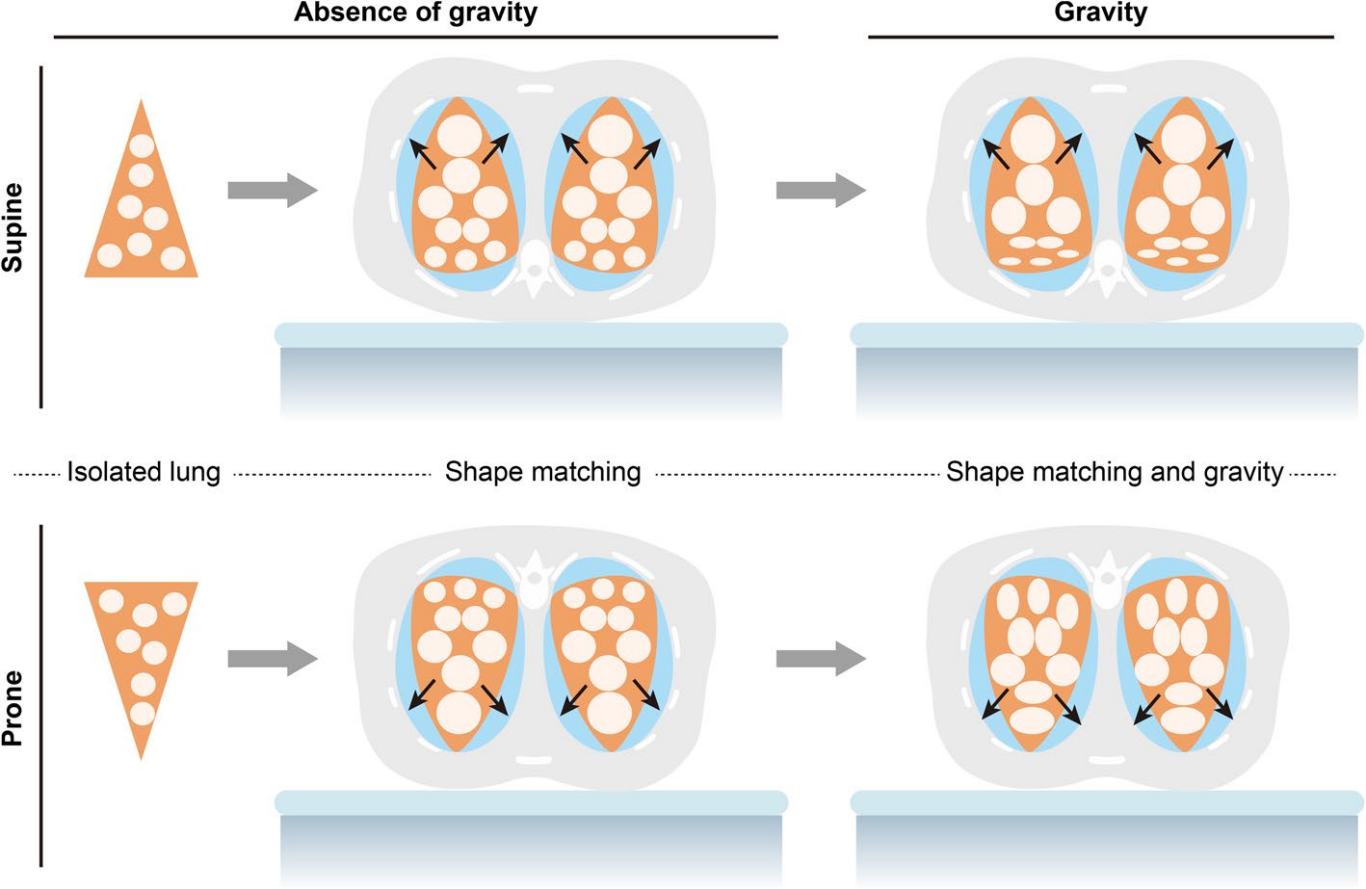
Thorax-lung shape mismatch. In this schematic representation of a transverse section of the thorax, the lung is represented as an orange triangle, while the pleural cavity is represented as a yellow oval. The isolated lung, in absence of gravity, can be thought of as a triangle, with all alveolar units of the same size. When the lung is placed into the thoracic cage, the apex of the cone stretches to adapt to the oval shape of the pleural cavity, which leads to an increase in size of the units in this area. When gravity is added, the units in the lower part of the lung tend to collapse due to the superimposed pressure of the units above. If the patient is then pronated, the hydrostatic pressure effect and the shape mismatch act in opposite directions, leading to a more homogeneous distribution of ventilation

**Fig. 7:**
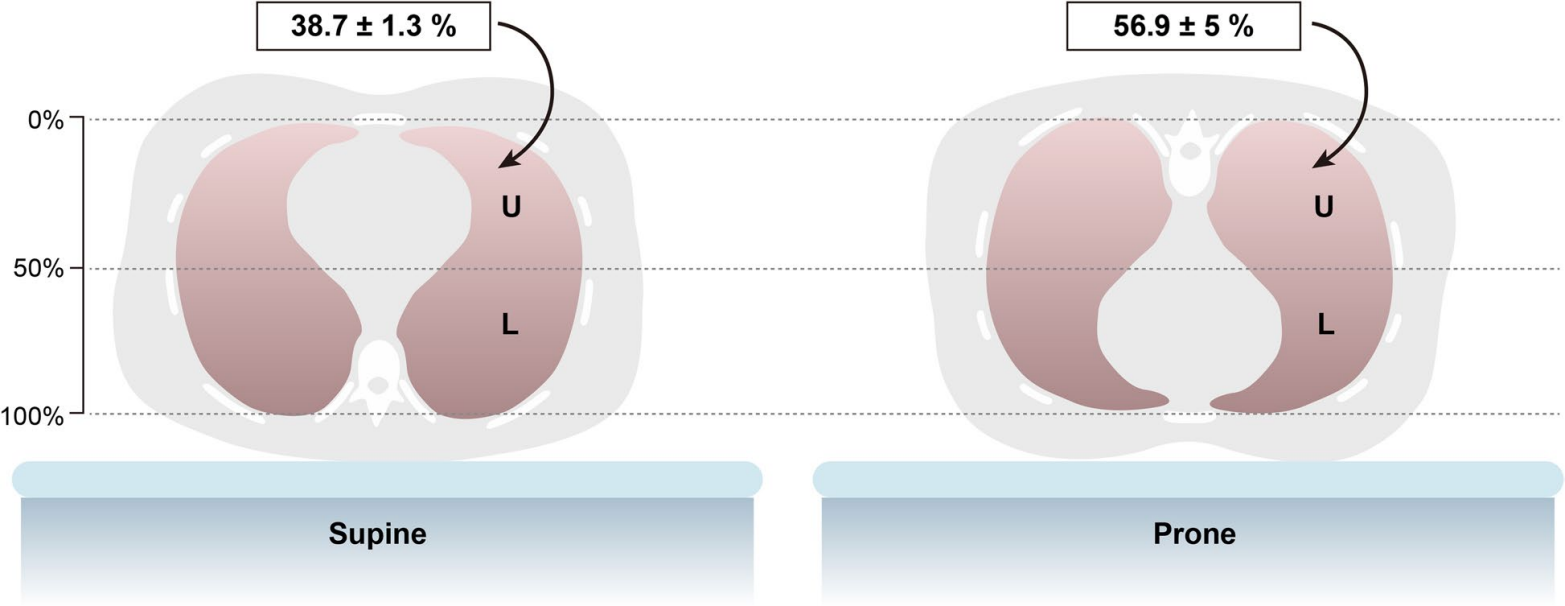
Lung mass distribution. Due to its anatomical conformation, most of lung parenchyma is located in the dorsal part of the thorax. The letter “U” indicates the *upper* part of the lung, while the letter “L” indicates the *lower* one. At 50% of the sternum-vertebra distance, the nondependent lung mass in the supine position is less than 40%, while it rises to almost 60% in the prone position

These conformational changes of chest wall and lung anatomy, when shifting from supine to prone, leads to changes of aeration into the lung parenchyma which may be summarized as follows:

i. **Homogenization of transpulmonary pressure.** The transpulmonary pressure (P_tp_), *i.e.* the difference between the airway pressure and pleural pressure (P_tp_ = P_aw_ – P_pl_), is the pressure responsible for lung distension. As pleural pressure varies according to a gravity-dependent gradient, the transpulmonary pressure progressively decreases going from the nondependent to the dependent regions, leading, in supine position, to a greater inflation/ventilation in the nondependent compared to dependent lung units [30]. In contrast, during prone position, the pleural and transpulmonary pressure gradients were found reduced, leading, in this position to a more homogeneous distribution of inflation/ventilation. This has been observed both in healthy and injured lungs (Fig. 5) [31, 32].
ii. **Thorax-lung shape mismatch.** The reduction in pleural pressure gradient is likely due to the interaction between gravity and the need for the lung and the chest wall to adapt their original shapes to occupy the same volume. In fact, the lung can be modeled as a cone having as base its dorsal surface, and the shape of the chest wall can be approximated the one of a cylinder (Fig. 6). In the supine position, gravity and the thorax-lung shape match act in an additive way: while the dorsal regions of the lung tend to collapse under the effect of gravity, the ventral regions tend to expand for the combined effects of gravity and the need to adapt to the shape of the chest wall. On the contrary, in the prone position, these two mechanisms balance each other: the dorsal regions tend to expand under the effect of gravity, while in the ventral regions the gravity-dependent tendency to collapse is counteracted by the tendency to expand to match the chest wall shape. Therefore, inflation of the dependent and nondependent pulmonary units is far more similar in prone than in the supine position [33, 34].
iii. **Lung mass distribution.** Due to the anatomical conic shape of the lung, the amount of tissue in the dorsal regions of the lung is greater than the one in the ventral part. In fact, at 50% of the sternum-vertebra distance, the dependent lung mass is about 60% of the total in the supine position, while it decreases to about 40% in the prone position. Consequently, fewer mass can collapse in the dependent zones in prone as compared to the supine position (Fig. 7) [32].

### Hemodynamics

Although the regional distribution of pulmonary blood flow remains unchanged in prone compared to supine position, several hemodynamics variations have been reported, as, during flat prone position, an increase of central venous pressure and central blood volume. This is due to the shift of splanchnic blood volume to the thorax related to the lowering of the trunk and to the compression of the splanchnic compartment [35–37]. This leads to a rise in pulmonary arterial occlusion pressure (PAOC) and, consequently, to a reduction of the transpulmonary vascular gradient (the difference between mean pulmonary arterial pressure and PAOC). Of note, high transpulmonary gradient defines vascular dysfunction, which is a major independent factor associated with mortality in ARDS [38]. Furthermore, a rise in PAOC might result in pulmonary vascular recruitment: as pulmonary venous pressure rises backwards, some pulmonary units could move from the West zone 2 to West zone 3, thus decreasing the dead space. This phenomenon, together with the decrease in P_a_CO2, the decrease in hypoxic vasoconstriction and the decrease in airway pressure due to a more homogeneous distribution of ventilation, accounts for a decrease in pulmonary vascular resistances [37]. These physiological changes explain the observed better function of the right ventricle, which benefits from both higher preload and decreased afterload. Indeed, Vieillard-Baron et al. [39] showed how the use of prone position leads to a decrease of right ventricular dilation and septal dyskinesia, together with a significant increase in cardiac index and a decrease in heart rate in a group of patients with acute cor pulmonale during ARDS. The increase in central venous blood can also result in an increase in cardiac output [40, 41]. Of note, Jozwiak et al. [37] analyzed a cohort of ARDS patients with dilated right ventricle and reported an increase of cardiac index associated with right ventricle unloading only when preload reserve was observed in supine position. Lastly, some more benefits provided by prone position on hemodynamics are a decreased incidence of cardiac arrest, as observed in the prone position group of the Proning Severe ARDS Patients (PROSEVA) study [42], and of arrhythmias, as shown by a systematic review [43].

Some minor side effects of prone position on hemodynamics have also been reported. They mainly consist in an increase in left ventricle afterload due to the compression of abdominal arteries, which could adversely affect left heart function, decrease splanchnic perfusion and increase renal vascular resistance. However, this effect is modest (the observed rise in mean arterial pressure is only of approx. 5 mmHg) [37] and no detectable alteration of either gastric intramucosal energy balance [41] or renal perfusion [40] has been reported.

## The clinical trials

Prone position is nowadays part of the standard of care for patients with moderate-severe ARDS. However, several RCTs were required before showing a significant benefit of prone position on outcome [42]. See Table 1 for summary.

**Table 1:** Clinical trials on prone position

	***Gattinoni *et al*. (NEJM, 2001)*** [44]	***Guérin *et al*. (JAMA, 2004)*** [45]	***Mancebo *et al*. (AJRCCM, 2006)*** [46]	***Taccone *et al*. (JAMA, 2009)*** [47]	***Guérin *et al*. (NEJM, 2013)*** [42]
*Patients (n)*	304	802	142	344	466
*Enrollment period*	1996–1999	1998–2002	1998–2002	2004–2008	2008–2011
*Inclusion criteria*	• P/F < 200 at 5 cmH_2_O PEEP or • P/F < 300 at 10 cmH_2_O PEEP	ARF from any cause with P/F < 300	ARDS according to the AECC definition	ARDS according to the AECC definition	P/F < 150 with PEEP ≥ 5 cmH_2_O and FiO_2_ ≥ 60%
*Average P/F ratio at enrollment*	127 ± 48	152 ± 59	161 ± 94 (supine) 132 ± 74 (prone)	113 ± 39	100 ± 25
*Average PEEP at enrollment*	10 ± 3	8 ± 3	8 ± 2	10 ± 3	10 ± 3.5
*Average tidal volume used (mL/kg)*	10.3 ± 2.8	11 ± 3	8.4 ± 1.7	8 ± 1.7	6.1 ± 0.6
*Stabilization after meeting inclusion criteria*	No	> 12–24 h	< 48 h	< 72 h	< 36 h
*Duration of prone position (hours/day)*	7 ± 1.8	8 (7.7 – 9.8)	17.7	18 ± 4	17 ± 3
*Number of days prone*	4.7	4 (2–6)	10 ± 10	8 ± 6	4 ± 4
*Mortality (prone* vs. *supine)*	• 50.7% *vs.* 48% at ICU discharge • 62.5% *vs.* 58.6 at 6 months	• 32.4% *vs.* 31.5% at 28 days • 43.3% *vs.* 42.2% at 90 days	• 43% *vs.* 58% at ICU discharge	• 31% *vs.* 32.8% at 28 days • 47% *vs.* 52.3% at 6 months	• 16% *vs.* 32.8% at 28 days • 23.6% *vs.* 41% at 90 days

*First randomized trial:* in the early 2000s, Gattinoni et al. [44] conducted a RCT on 304 patients, aiming to determine whether this technique, already widespread at that time, could improve outcome. Patients were assigned either to usual care or cyclic pronation, with proning sessions of at least 6 h per day over a period of 10 days. Patients were evaluated each morning while laying supine and, if they fulfilled acute lung injury (ALI)/ARDS criteria, they were proned. Mortality at 10 days, at discharge from ICU and at six months did not differ significantly between the two groups. This result was somehow unexpected, as patients in the pronation group showed a significant increase in oxygenation, with a median increase in P_a_O_2_/F_i_O_2_ by 44 mmHg and an increase in P_a_O_2_/F_i_O_2_ by more than 10% in 73% of the study population. The variability in response among individuals remained high. When adopting the supine position again, part of the improvement in oxygenation due to prone position was lost. However, these patients showed better P_a_O_2_/F_i_O_2_ ratios as compared to control group, even when evaluated in the supine position. A question arose: why did prone position not work? A partial explanation was given by authors themselves, who hypothesized that the potential benefits of prone position may become evident only when the technique is adopted either in the most severe patients or for longer periods of time. Indeed, in the same study, a *post-hoc* analysis conducted on patients with lower P_a_O_2_/F_i_O_2_ ratio (P_a_O_2_/F_i_O_2_ < 88) showed improved survival at 10 days (23% vs. 47%). The same positive benefit was also present in the quartile of patients with the highest SAPS II scores. Mortality, instead, remained the same in the longer run (no difference at ICU discharge). The authors concluded that prone position could have either delayed the inevitable outcome of death in these more severe patients, or, alternatively, that the duration of pronation was not sufficient to assure a long-lasting reduction in mortality. Interestingly, few years later the same authors published a secondary analysis [24] using the data of the aforementioned study, aiming to assess if gas exchange improvement due to prone position could be predictive of an improved outcome. The study highlighted how the “responders” (patients whose P_a_O_2_/F_i_O_2_ ratio improved after pronation) had, surprisingly, the same outcome as “non-responders”. This was one of the first studies suggesting the potential benefits of prone position on outcome might not rely on improved oxygenation, but rather on a less injurious mechanical ventilation, due to a more homogenous distribution of stress and strain within the respiratory system. Giving these premises, a further investigation was necessary.

*Second randomized trial:* few years later, Guérin et al. published on JAMA [45] the results of a RCT, in which 791 patients with acute hypoxemic respiratory failure from all causes were assigned either to cyclic pronation sessions (8 h/day) or to standard care. Despite the huge number of subjects involved, no differences in 28 and 90 day mortality were found. These “negative” results, however, must be cautiously interpreted, as only about 50% of patients in both arms met the criteria for ALI/ARDS. Mortality in this heterogeneous population may depend on several factors, some of which independent from the type of mechanical ventilation applied.

*Third randomized trial:* in order to address the questions left unanswered by previous trials, in 2006 Mancebo et al. [46] published a study in which ARDS patients, 48 h after intubation and start of mechanical ventilation, were randomized either to continuous prone position (20 h/day) or to standard care. Ventilator settings and weaning procedures were standardized in the two groups. The study was designed to show a difference in mortality of 20% and it required 200 patients. Unfortunately, the authors managed to enroll only 142 participants; nonetheless, the results were sound. The patients in the interventional arm of the study were kept prone for an average of 17 h/day, over an average of 10 days. As expected, prone patients needed lower F_i_O_2_ and positive end expiratory pressure (PEEP) levels, which allowed also to keep slightly lower plateau pressures. Mortality at ICU discharge was 58% in the supine group and 43% in the prone one; the results did not reach classical statistical significance, likely due to the lower-than-planned number of patients enrolled. Therefore, despite the non-significant results, the study showed an 15% absolute and a 25% relative reduction in mortality. Furthermore, the results of a multivariable logistic regression indicated the random allocation to the supine position was a risk factor for mortality, with an OR of 2.53. In conclusion, the study by Mancebo et. al was the first one evaluating the use of early and continuous prone position in a population of ARDS patients; the results, despite non-significant, were and still are of invaluable interest for the scientific community.

*Fourth randomized trial:* on the wave of the study performed by Mancebo et al., the Prone-Supine Study Group (the same authors of the paper by Gattinoni et al. [44]) organized a second multicenter RCT – the Prone Supine II (PSII) [47] – overcoming the recognized limitations from 2001 trial. As in Mancebo’s, patients in the PSII study were randomized to receive prolonged pronation within 72 h from ARDS diagnosis or standard care. A total of 342 patients was enrolled, prospectively stratified in a group with moderate hypoxemia (P_a_O_2_/F_i_O_2_ ratio between 100 and 200 mmHg) and severe hypoxemia (P_a_O_2_/F_i_O_2_ ratio < 100 mmHg). Patients underwent a mean of 8.4 ± 6.3 pronation sessions, with an average duration of 18 ± 4 h. The primary outcome, mortality at day 28, was not different in the two groups (31% *vs.* 32.8%, RR 0.97, *P* = 0.72), while, in patients with more severe hypoxemia, mortality was 37.8% in the prone group *vs.* 46.1% in the control group (RR 0.87, *P* = 0.31). Similar results were obtained also for the 6-month mortality: in the most severe patients, authors were able to show a 10% mortality reduction in the prone group compared to controls (RR 0.78, *P* = 0.19). Overall, the results of the study were consistent with the prior findings, yet still unable to demonstrate clearly the benefits of prone position. The authors concluded that its use in an unselected ARDS population may not be indicated as a treatment, but for the most severe patients, it must be further investigated, as the benefits could outweigh the complications, given the strong pathophysiological background and the findings of the previous studies.

*Fifth randomized trial:* in 2013, Guérin et al. published the results of the PROSEVA study on the New England Journal of Medicine [42]. It was a multicenter RCT involving 26 ICUs in France and one in Spain. In order to translate the known physiological benefits of prone position into a better outcome for patients, the study provided very clear enrollment criteria, aiming to focus only on the most severe patients. Inclusion criteria were: mechanical ventilation for less than 36 h, a P_a_O_2_/F_i_O_2_ ratio less than 150, PEEP of at least 5 cmH_2_O and a F_i_O_2_ equal or greater than 0.6. The confirmation of these criteria was checked after 12–24 h of mechanical ventilation. Particularly, including a short stabilization period after the initiation of mechanical ventilation allowed to select only the subjects with the most compromised lungs. Of the 474 randomized patients, 466 were included in the final analysis (supine = 229, prone = 237) and in both groups the main cause of ARDS was pneumonia (58.1% supine *vs.* 62.4% prone). The characteristics at baseline were not different in the two groups and enclosed a quite severe population of ARDS patients. Indeed, the mean P_a_O_2_/F_i_O_2_ ratio at enrollment was 100 ± 20 in the supine group and 100 ± 30 in the prone group, with a mean PEEP level of 10 cmH_2_O and a mean F_i_O_2_ of 79% in both groups. Protective ventilation with a tidal volume of 6 ml/kg PBW was used, resulting in mild respiratory acidosis (P_a_CO_2_ 52 ± 32 mmHg in the supine group *vs.* 50 ± 14 mmHg in the prone group; pH 7.30 ± 0.10 in both groups). Patients assigned to the pronation arm of the study underwent the first session 34 ± 24 h after intubation; the mean number of sessions per patient was 4 ± 4, and the sessions lasted an average of 17 ± 3 h. In the first week after randomization, respiratory mechanics and gas exchange were measured daily. As expected, the pronation arm showed lower PEEP and F_i_O_2_ values, higher P_a_O_2_/F_i_O_2_ ratios and slightly better respiratory mechanics with lower plateau pressures. Crossover from the supine to the prone group was a rare event and occurred only in 17 cases. This was due to strict criteria, which allowed crossover only for patients with refractory hypoxemia (P_a_O_2_/F_i_O_2_ < 55 with F_i_O_2_ of 0.1), in spite of PEEP optimization and use of adjunctive therapies, such as inhaled nitric oxide or intravenous almitrine. Notably, their use was higher in the supine group than in the prone one. Mortality was evaluated at 28 and 90 days. Patients assigned to pronation group had 16% mortality rate at 28 days compared to 32.8% of the supine group (HR 0.39, *p* < 0.001); the same benefit was also present at 90 days (23.6% *vs.* 41% mortality, HR 0.44, *p* < 0.001). Eventually, prone patients were successfully extubated more often and had more ventilator free days. The study by Guérin is a landmark in the literature of ARDS treatment. The severity of the population studied, the precocity of the intervention and the prolonged application of the prone position were all factors which contributed to the positive results of the study and allowed the scientific community to define once for all, until proven otherwise, who are the patients prone position is indicated for and which “dose” (length of treatment) is necessary to improve outcome.

### Why should prone position affect outcome

It must be always remembered that whatever respiratory technique applied in ARDS just aims to buy time necessary for the recovery from the disease leading to ARDS while maintaining gas exchange compatible with life. Therefore, when we compare different techniques, being the benefits the same (*i.e.*, adequate gas exchange), we are actually comparing the harms associated with them. Indeed, all the large trials, such as EOLIA and ARMA, did not find any relationship between oxygenation and outcome [48, 49]. Indeed, what matters is the cost of different technique to provide the same benefit. In this framework, the reasons why prone position may provide benefit by decreasing the ventilation harm may be summarized as follows:

- The prone position is always associated with a more homogeneous distribution of inflation throughout the lung parenchyma. This implies that whatever ventilation applied in prone is more homogenously distributed than in supine, avoiding/dumping focuses of harmful stress and strain applied to the extracellular lung matrix, *i.e.* the primary cause of ventilator-induced lung injury (VILI) [50]. The effects of prone position on lung protection were described in studies conducted both on animal models [51, 52] and on patients with ARDS [53, 54]. In addition, the use of the prone position is associated with decreased lung inflammation in patients with ARDS [55], resulting in a reduction of biotrauma [56]. These mechanisms of VILI reduction are attributed to the mortality benefit of prone position reported in the PROSEVA trial as well as in other observational studies in ARDS and COVID-19 ARDS [57].
- The better oxygenation in prone position implies the possible use of lower F_i_O_2_, thus avoiding and limiting whatever problem of oxygen toxicity [58].
- Prone position facilitate the mobilization and drainage of secretions [59], with significant reduction in the risk of ventilator-associated pneumonia (VAP) [47, 60–62].

## Clinical application

### Indications

As a consequence of the results of the PROSEVA study, prone position is currently part of the standard of care for patients suffering from moderate-severe and severe ARDS (*i.e.*, P_a_O_2_/F_i_O_2_ < 150 mmHg with at least 5 cmH_2_O PEEP) [63], as a technique to reduce mortality.

In contrast, its use in patients with mild-moderate and mild forms of the syndrome (P_a_O_2_/F_i_O_2_ > 150 mmHg) is questionable. So far, no randomized trials have succeeded in demonstrating a mortality reduction in patients with ARDS and P_a_O_2_/F_i_O_2_ > 150 mmHg. Nevertheless, both randomized studies and meta-analyses conduced on patients with mild or moderate ARDS were markedly underpowered for a mortality endpoint. Thus, they are not sufficient to prove that prone position is not beneficial in this conditions, and additional studies are needed [64]. Moreover, it is important to underline that prone ventilation must not be considered as a rescue therapy anymore. In fact, as its main benefit rely on VILI prevention, it should be used as a routine procedure, and should be initiated early in the course of ARDS rather than in a late phase, when VILI has already been established [57, 65].

### Contraindications

Absolute contraindications to prone positioning are limited to spinal instability [65].

Other relative contraindications have been proposed, including unstable pelvic or long bone fractures, rheumatoid arthritis affecting the atlanto-occipital joint, open abdominal wounds, severe burns, late-term pregnancy, increased intracranial pressure, and severe hemodynamic instability. In these conditions, higher risks of complications following prone positioning are expected, but they should be balanced with the known benefits provided by a potentially life-saving treatment. Moreover, some precautions can be taken to minimize the risk of complications. For example, before proning, fractures should be stabilized, while wounds and burns can be properly dressed. Similarly, patients with rheumatoid arthritis affecting the atlanto-occipital joint can be proned after placing of a neck collar. Pregnant women can undergo prone positioning provided that attention is given to limit abdominal and pelvic compression and continuous fetal heart tones monitoring is used. Intracranial hypertension, which could worsen during prone position due to the partial obstruction of cerebral venous drainage, it ought to be controlled by measuring intracranial pressure, using it as a guidance to correctly position the head and neck. Concerning hemodynamic instability, it is true that all patients were hemodynamically stable at the time of inclusion in the PROSEVA study; on the other hand, as previously explained, prone position is not related per se to hemodynamic impairment, while it could even improve hemodynamics. Therefore, a careful risk-benefits assessment should be carried out before performing prone procedure, considering the characteristics of each single patient and the expertise of the medical and nursing team.

Finally, it is worth noting that obesity, while making prone procedure more challenging, is not to be considered a contraindication. Indeed, prone position has been proved to be beneficial in obese anesthetized subjects by improving pulmonary function, lung compliance and oxygenation [66]. Furthermore, prone position can be also used with patients on extracorporeal circulation (extracorporeal membrane oxygenation (ECMO) or extracorporeal CO_2_ removal (ECCO_2_R)) or ventilated through tracheotomy tubes, provided cannulas/tubes are handled with care in order to avoid undue torsion.

### Prone position maneuver

There are several ways to move a patient from supine to prone position, and most centers have developed their own local protocol to perform prone procedure, in order to minimize risks for both patients and staff (back injuries). An algorithm describing in detail how to perform safe prone position maneuver has been proposed by Messerole et al. [67].

In general, prone position maneuver should be performed by at least 3 people, one at each side of the bed and one (usually a physician) at the head of the bed, taking care of the endotracheal tube and central lines. When performed in ECMO or ECCO_2_R patients, one more person is needed to look after the extracorporeal circuit. During prone position maneuver, the standard monitoring should include pulse oximetry and invasive arterial blood pressure. After increasing FiO2 to 100%, the patient is carefully turned in lateral decubitus (either left or right, generally turning his/her face toward the ventilator) and then to prone position, with head turned to the left or right to minimize facial trauma and arms in a comfortable position to prevent brachial plexus injuries. Then, endotracheal tube and central venous catheters must be secured, after checking their correct position and function. Thereafter, the patient should be tilted into reverse Trendelenburg to reduce the risk of esophageal reflux and/or aspiration. Eyes occlusion is recommended to prevent conjunctivitis and corneal ulcerations, as well as padding for body areas in contact with the bed (*e.g.*, eyes, cheeks, breasts, anterior iliac spines).

Prone position usually does not require any additional monitoring. Electrocardiographic leads might be placed on the back to reduce damage to chest skin. Ventilation can be adjusted, in order to achieve protective ventilation (low tidal volume around 6 mL/kg predicted body weight, plateau airway pressure less than 30 cmH_2_O) [68] and adequate P_a_CO_2_. More frequent endotracheal aspiration may be needed, because a larger amount of airway secretions may deposit into the endotracheal tube. In order to prevent facial skin breakdown, face should be turned from one side to the other every 2–4 h. The utilization of thoraco-pelvic supports did not prove effective [69].

Prone position is generally accompanied by continuous neuromuscular blockade [42]. As a consequence, deep sedation is also often necessary.

### Duration and stopping criteria

Since the benefits of prone position relate to the decreased damage due to mechanical ventilation, the longer the time spent daily in the prone position, the higher is the lung protection [70, 71]. Unfortunately, the exact time threshold required to optimize benefits of prone position is still unknown. Recently, a meta-analysis conducted by Munshi et al. [72] found reduced mortality when prone position was applied for at least 12 h daily, while the PROSEVA study aimed to maintain prone position for at least 16 consecutive hours [42].

The usual criteria for stopping prone ventilation and turn the patient back to the supine position are deterioration of P_a_O_2_/F_i_O_2_ ratio by more than 20% as compared to supine or the occurrence of a life-threatening unscheduled complication (*e.g.*, nonscheduled extubation, main-stem bronchus intubation, hemoptysis, sudden decrease in oxygenation, cardiac arrest).

In the PROSEVA study, cycles of prone positioning were repeated every day, even if the previous prone session did not show any improvement in oxygenation, until improvement in oxygenation in supine position was reached (PaO_2_:FiO_2_ ≥ 150 mmHg with PEEP ≤ 10 cmH_2_O and an FiO_2_ ≤ 60%, at least 4 h after the end of the last prone session). The mechanisms explaining the outcome improvement are complex and, thus, a lack in oxygenation improvement should not be considered a criterion to stop proning sessions [57].

### Side effects

The most severe and dangerous reported complications concern airways control (*i.e.*, accidental extubation, selective bronchial intubation and endotracheal tube obstruction) or device displacement (*e.g.*, of thoracic tubes, vascular catheters or extracorporeal cannulas). Other referred adverse effects consist in transient hypotension and/or desaturation (mainly occurring during the proning maneuver), vomiting, arrhythmias and cardiac arrest. Moreover, frequently observed complications were pressure ulcers (more frequent as compared to the supine position [73]), facial and ocular edema due to vascular stasis, retinal damage and brachial plexus neuropathy.

Interestingly, the PROSEVA study showed no difference in adverse effects between supine and prone groups, except for cardiac arrest (more frequent in the supine group) [42]. This might suggest that ICU team’s experience in managing prone position could prevent the development of most complications.

A series of recent meta-analysis reported no differences between prone and supine positions for risks of unplanned central catheter removal, unplanned extubation, barotrauma, pneumothorax, cardiac arrest, brady- or tachyarrhythmias, or ventilator-associated pneumonia. Conversely, there were increased risks of endotracheal tube obstruction and pressure ulcers in the prone group [72, 74].

Furthermore, a recent international observational study reported a very low rate of complications due to prone position as compared to previous clinical trials [75], which might partially be due to improvement in clinical practice.

## Prone position in COVID-19

During COVID-19 pandemic, due to the increasing number of patients with Sars-Cov2-related acute respiratory failure, the use of prone position rose to prominence to such an extent that the Surviving Sepsis Campaign subcommittee recommended it among the available symptomatic treatments [76]. Early phase COVID-19 patients typically present at the physicians’ attention with a clinical picture characterized by the presence of severe hypoxemia in association with well-preserved lung mechanics and in absence of widespread pulmonary consolidations [77]. The adoption of the prone position has been one of the first strategies attempted to reverse hypoxemia. How the employment of this decubitus became customary is reported by large prospective cohorts studies, indicating a rate of adoption of prone position around 70% in COVID-19 patients, while its pre-pandemic rate was estimated to be only 20% [78]. The effectiveness of pronation is acknowledged as an improved oxygenation corresponding to an increase in PaO_2_ or PaO_2_/FiO_2_ ratio – during or after the maneuver – equal to or greater than 20% of baseline levels. The patients showing this amelioration are referred to as “responders”.

Regarding the determinants accountable for this improvement in COVID-19 patients, they are thought to be likely related to the degree of ventilation/perfusion mismatch and to the amount of atelectasis and consolidations developed in the lung parenchyma. Nevertheless, the precise mechanisms underlying the effectiveness of pronation in COVID-19 pneumonia are not completely understood. Moreover, one relevant issue about prone position regards its short-lasting effect. Indeed, the abovementioned improvement in oxygenation has been found quickly revertible once the patients return back to supine position, even in those undergoing long sessions of awake pronation [79, 80]. The lack of significant amount of dorsal atelectasis, whose recruitment with unmodified perfusion is the primary mechanism of improved oxygenation in classical ARDS, suggests a different mechanism in Covid-19 patients, *i.e.* a preponderant role of the redistribution of perfusion during prone position. Due to gravity, the flow is redirected predominantly to the pulmonary ventral regions, as a result of vascular tone loss and dysregulation, events frequently occurring in COVID-19 patients [81]. The response to pronation in more advanced states of the disease (1–2 weeks) appears to depend on the amount of atelectasis and/or consolidations present in the lung parenchyma, which increase with time. If atelectasis (*i.e.* pulmonary units empty but collapsed) prevails over consolidations (*i.e.* pulmonary units full of material but gasless), the net effect of prone position will be a rise in P_a_O_2_. In contrast, if consolidations outweigh atelectasis – as it likely occurs in the late phase of Sars-Cov-2 pneumonia – the response to pronation will be rather ineffective. Of note, the possible presence of new anastomoses established between the bronchial circulation and the pulmonary one can make the whole picture more elaborate [82]. Even in case of a pathophysiological mechanism differing from the aforementioned ones, in a recent study the percentage of intubated prone position responders was approximately 80% both in COVID-19-related ARDS (the so called C-ARDS [83]) and in classical ARDS forms [84].

### Awake prone position

#### Indications and advantages

The lack of ICU resources during the first wave of the pandemic lead to use prone position in awake, spontaneous breathing COVID-19 patients to correct hypoxemia. Its use has been often associated with high flow nasal cannula (HFNC), continuous positive airway pressure (CPAP) or non-invasive ventilation (NIV). Before the widespread of Sars-Cov-2 infection, the employment of awake prone position was unusual, and this is evidenced by the scarce number of studies focusing on this subject – only a few case reports [85] and one retrospective analysis [86] – though all demonstrating the feasibility of the maneuver and its beneficial action in terms of oxygenation. The same results are inferable from the majority of studies conducted since the beginning of the pandemic [79, 87, 88]. The pathophysiological mechanisms underlying awake pronation are perhaps the same as the ones occurring in intubated patients, including the advantages other than gas exchange improvement. Possible advantages are here below summarized:

- Firstly, awake prone position could be helpful in reducing the patient self-induced lung injury [89]. In fact, COVID-19 patients often present with hypoxemia and strong inspiratory efforts worsen by high respiratory rates. These lead to an increased work of breathing, assessable with physical examination, and to an excessive stress and strain in lung parenchyma. A recent study has, indeed, demonstrated the efficacy of awake prone position in combination with CPAP in reducing the work of breathing, possibly lowering the risk of progression to critical illness, and subjective dyspnea in all the patient experiencing it [90].
- Awake prone position may delay or even avoid intubation, thus decreasing the ICU overload. Yet, data regarding the effect of awake prone position on intubation are controversial. A RCT showed no differences in the rate of endotracheal intubation at 30 days after randomization in patients treated with awake prone position and controls [91]. On the contrary, a meta-analysis of 29 studies, of which 10 were RCTs, pointed out a reduction of intubation rate, mostly in patients requiring high oxygen delivery and in ICU inpatients [92]. The reasons for these discrepancies are still unclear.

It is worth noting, however, that no outcome benefit, has been found clearly associated with the use of prone position in awake patients. It is tempting to speculate that it is due to the lack of the pre-requisite needed for the prone position to improve outcome, *i.e.*, remarkable stress and strain maldistribution associated with presence of atelectasis and consolidation, usually modest in awake COVID-19 patients.

#### Timing and duration

Regarding the optimal timing of awake pronation sessions, there are currently no straightforward recommendations. However, taking into account the brief duration of oxygenation improvement, the initiation of sessions should be performed right after hospitalization. A recent study showed a decrease in 28 day mortality in non-intubated patients when they adopted the so called “early awake prone position”, *i.e.* the start of awake prone position in first 24 h after the initiation of HFNC [93]. In addition to the prompt decision making, it is indispensable to close monitor patients’ response to pronation, in order to early detect non-succeeders, namely those who could depend on endotracheal intubation to survive. Indeed, a delayed transition to invasive mechanical ventilation could be detrimental to their outcome [94].

The best predictor associated with oxygenation improvement in awake COVID-19 patients requiring HFNC were: prone position duration > 8 h/day, respiratory rate ≤ 25 bpm at enrollment, increase in respiratory-oxygenation (ROX) index > 1.25 after the first awake prone position session and a decrease in lung ultrasound score ≥ 2 [95]. Six hour/day duration was found as the minimum length of awake prone position to reduce the risk of resorting to endotracheal intubation. Actually, extending session length to 8 h could be even more effective, as it seems to lower hospital mortality [96]. The duration of the maneuver is awake patients may be problematic. Indeed, a meta-analysis of eight studies indicates an intolerance rate among patients equal to 10.3% [97], primarily due to poor comfort [98]. This may be reduced by new devices and by a proper explanation of the technique to the patients [99]. Positions alternative to classical prone have been tested (*e.g.*, Rodin’s position [100], dolphin position [101], reverse Trendelenburg [102], alternating prone [103]).

#### Side effects

Prone position in awake patients is easier to perform than in intubated patients. Indeed, less personnel, time and resources are required, due to the collaboration of patients who can even perform the maneuver by themselves [104]. Additionally, there are lower risks of vascular accesses and devices displacement. Other rare side effects reported on awake pronation session include pressure ulcers (*e.g.*, facial), nerve compressions (brachial plexus injury), crush injury, venous stasis (facial edema), diaphragm limitation, retinal damage, vomiting and transient arrhythmias [105].

## Conclusions

After decades from the first clinical application of prone position, we know the physiological changes associated with this technique, their consequences on mechanical effects of ventilation and the possible benefits associated with the maneuver. In summary, prone position makes the lung parenchyma more homogeneous. This results in a more homogenous distribution of ventilation and of mechanical stresses and strain, the putative causes of VILI. The pre-requisite to observe significant benefit on mortality is that a significant degree of baseline stress and strain maldistribution, as associated with moderate-severe and severe ARDS. The oxygenation improvement is the most common effect of prone position, although not directly associated with mortality. In general, however, regardless strict indications or recommendations, the bulk of available data indicates that, whatever is the harm induced in the lung by the ventilation, mechanical or spontaneous, is lower in prone than in supine. 

## Data Availability

Not applicable.

## Abbreviations

ARDS: Acute Respiratory Distress Syndrome
FRC: Functional residual capacity
RCT: Randomized controlled trials
PaCO2: Arterial partial pressure of CO_2_
PaO2: Arterial partial pressure of O_2_
P_tp_: Transpulmonary pressure P_tp_
PAOC: Pulmonary arterial occlusion pressure
ALI: Acute lung injury
ICU: Intensive care unit
PEEP: Positive end expiratory pressure
PROSEVA: Proning Severe ARDS Patients
PSII: Prone Supine II
VILI: Ventilator-induced lung injury
VAP: Ventilator-associated pneumonia
ECMO: Extracorporeal membrane oxygenation
ECCO_2_R: Extracorporeal CO2 removal
HFNC: High flow nasal cannula
CPAP: Continuous positive airway pressure
NIV: Non-invasive ventilation
ROX: Respiratory-oxygenation
